# A comparative analysis of molecular genotypes of *Mycobacterium tuberculosis* isolates from HIV-positive and HIV-negative patients

**DOI:** 10.3389/fcimb.2022.953443

**Published:** 2022-10-12

**Authors:** Jitendra Singh, Niti Singh, Gayatri Suresh, Rahul Srivastava, Upasna Aggarwal, Digamber Behera, Murali Munisamy, Anvita Gupta Malhotra, Sarman Singh

**Affiliations:** ^1^ Division of Clinical Microbiology and Molecular Medicine, All India Institute of Medical Sciences, New Delhi, India; ^2^ Translational Medicine Centre, All India Institute of Medical Sciences, Bhopal, India; ^3^ Department of Microbiology, National Institute of Tuberculosis and Respiratory Diseases (NITRD), New Delhi, India; ^4^ Department of Pulmonary Medicine, PGIMER, Chandigarh, India; ^5^ Department of Microbiology, All India Institute of Medical Sciences, Bhopal, India; ^6^ Medical Science and Engineering Research Centre, Indian Institute of Science Education and Research, Bhopal, India

**Keywords:** spoligotyping, genotypes, HIV, *Mycobacerium tuberculosis*, molecular epidemiology

## Abstract

**Setting:**

Tuberculosis Research Laboratory, Division of Clinical Microbiology and Molecular Medicine, Department of Laboratory Medicine, All India Institute of Medical Sciences, and the National Institute of Tuberculosis and Respiratory Diseases (NITRD), both situated in New Delhi.

**Objectives:**

We aimed to identify the distribution of various genotypes of *M. tuberculosis* among HIV-positive and HIV-negative patients suspected of having Tuberculosis, seen at the National Institute of Tuberculosis and Respiratory Diseases, New Delhi, which is a tertiary care dedicated TB hospital.

**Patients and methods:**

Genotyping by Spoligotyping and 24 loci MIRU-VNTR was performed and analyzed using SITVITWEB and MIRU-VNTRplus. Drug susceptibility patterns were also analyzed.

**Results:**

A total of 503 subjects who were PTB/EPTB suspected were recruited and 287 were culture positive. Among them, 276 had growth of *Mycobacterium tuberculosis* (MTB) and in 11 patients non-tuberculous mycobacteria (NTM) were grown. The isolation rate of NTM was predominantly from HIV positive [10 of 130 (7.6%)] patients. Of the total isolates of MTB, 156 (56.5%) were from HIV negative patients and 120 (43.5%) were from HIV positive patients. All 276 *M. tuberculosis* isolates were genotyped and tested for drug susceptibility patterns. The CAS genotype was most predominant [153 (55.4%)], followed by Beijing lineage [44 (15.9%)], East African India [25 (9.1%)] and others [54 (19.6%)]. Beijing genotype was significantly more common in HIV positive patients (22.5%) than in HIV negative patients (10.9%). In MIRU-VNTR analysis, clustering was found to be more frequent in CAS strains irrespective of HIV status. In the HIV positive group, spoligotyping could differentiate various genotypes in 90% of isolates and MIRU-VNTR analysis in 84.2% of isolates. The clustering of various MTB strains was more associated with drug resistance.

**Conclusion:**

The Beijing lineage was predominant in HIV-TB coinfected cases, even though the Central Asian Strain (CAS) was overall more predominant in the region.

## Introduction

Tuberculosis (TB) and Human immunodeficiency virus (HIV) infections are lethal chronic infections and are among the leading causes of mortality globally. In 2019, an estimated 1.2 million TB deaths occurred among HIV-negative people and 208,000 deaths amongst HIV-positive people (Global tuberculosis report, 2020). As per a WHO 2020 report, 69% of notified TB patients had documented HIV test results, as compared to 64% in 2018. The burden of HIV-associated TB is the highest in the WHO African region, where 86% of TB patients had a documented HIV test result. Globally, 88% of the TB-HIV co-infected patients were on antiretroviral therapy (ART), while this proportion was 95% in India (WHO, 2016).

Although both TB and HIV are deadly by themselves, they have an additive pathogenic influence when they come together, thereby making the HIV-TB co-infection a “double trouble” for society (Munawwar and Singh, 2012). Coordinated pathogenesis of HIV and *Mycobacterium tuberculosis* poses a major medical challenge as HIV provides an enormous opportunity for *M. tuberculosis* to multiply rapidly within the intracellular setting, and *M. tuberculosis* provides a favorable environment for HIV to replicate unhindered *via* TB-associated protein malnutrition and increased depletion or non-activation of T lymphocytes (Friedland et al., 2007; Munawwar and Singh, 2012).

Molecular genotyping reveals the different levels of genetic polymorphisms of *M. tuberculosis* and is applicable in monitoring disease transmission, detecting outbreaks, and confirming laboratory cross-contaminations and clonal spread of dominant clones. Under certain circumstances, it is used to distinguish between members of the *M. tuberculosis* complex that have critical clinical importance (Allix-Béguec et al., 2010). Due to its clonal structure (Sreevatsan et al., 1997), the comparative genotype analysis of MTCs from different human populations can give unique insights into the dissemination dynamics and evolutionary genetics of this pathogen; therefore, spoligotyping has been used to understand the emerging problem of multidrug-resistant (MDR) TB and the virulence of certain epidemic strains of *M. tuberculosis* (for example; the Beijing strain), as well as to better understand the epidemiology of TB and TB-HIV co-infection (Cadmus et al., 2011).

Spoligotyping is based on polymorphism in the direct repeat (DR) locus and is used as a primary genotyping method in combination with molecular typing methods based on variable number tandem repeats (VNTRs) of the 24 loci DNA elements known as mycobacterial interspersed repetitive units (MIRU) (Supply et al., 2006). MIRU-VNTR in combination with spoligotyping has become a striking alternative to the traditional IS6110-RFLP fingerprinting method, with maximum discriminatory power (Pitondo-Silva et al., 2013).

Information on the genotypes of *M. tuberculosis* isolated from India, in comparison with those obtained globally, are important for understanding the global spread and phylogeographical specificity of the predominant circulating clones of tubercle bacilli. In addition, *M. tuberculosis* molecular genotyping data obtained from India so far have not addressed the issue of HIV-TB co-infected patients, a subpopulation involved in the spread of the disease in Asia in general and India in particular (Gutierrez et al., 2006; Sharma et al., 2008; Thomas et al., 2011; Vadwai et al., 2012; Singh et al., 2015). We have previously analyzed the molecular genotypes of *M. tuberculosis* among extrapulmonary and pulmonary tuberculosis patients irrespective of their HIV status (Sankar et al., 2013; Singh et al., 2015). Here, we aimed to investigate the spoligotyping and 24 loci MIRU-VNTR based population structure of *M. tuberculosis* clinical isolates from HIV negative and MTB positive patients and HIV-TB co-infected patients, and to compare the patterns obtained with those available in the SIT-VIT web database of the Pasteur Institute of Guadeloupe (Demay et al., 2012).

## Materials and methods

### Patient inclusion

This study was performed at the Tuberculosis Research Laboratory, Division of Clinical Microbiology and Molecular Medicines, All India Institute of Medical Sciences (AIIMS), New Delhi. The total duration of the study was three years from April 2012 to March 2015. All the patients were recruited at the National Institute of Tuberculosis and Respiratory Diseases (NITRD), New Delhi. As a standard protocol in India, all TB suspected cases are tested for HIV as per the standard guidelines issued by the National AIDS Control Organization and WHO. The inclusion criteria were all HIV-positive patients suspected of concomitant tuberculosis infection and clinically suspected patients with pulmonary tuberculosis (PTB) and/or extra-pulmonary tuberculosis (EPTB). Informed consent was obtained from all the recruited cases. However, we excluded patients taking anti-tuberculous treatment (ATT) for more than two weeks at the time of recruitment, patients under immune-suppressive treatment, and pregnant female patients. This study was approved by the Institutional Ethics Committees of the All India Institute of Medical Sciences, and the National Institute of Tuberculosis and Respiratory Diseases, New Delhi.

### Sample processing and identification

The clinical samples were transported on a daily basis to the Clinical Microbiology Laboratory at the All India Institute of Medical Sciences, New Delhi, where these were inoculated into BACTEC MGIT™ 960 as described earlier (Sankar et al., 2013). Flashed positive cultures were confirmed as *M. tuberculosis* by species specific *multiplex* PCR (m-PCR), which amplifies *hsp65*, *esat6*, and *its* regions of the mycobacterial genome (Sankar et al., 2013).

### Genotyping

DNA from the cultures was isolated as previously described (Sankar et al., 2013). After checking the quality of the DNA, spoligotyping was performed using a commercially available kit (Mapmygenome India Limited) as per the manufacturer’s instructions (Thomas et al., 2011; Vadwai et al., 2012; Singh et al., 2015). Twenty-four loci MIRU-VNTR was performed by PCR amplification of individual loci using specific primers, as described previously (Supply et al., 2006).

### Drug susceptibility test

The susceptibility pattern to Isoniazid (INH), Rifampicin (RIF), Streptomycin (SM), and Ethambutol (EMB) was determined using the BACTEC MGIT™ 960 (Becton- Dickinson, Sparks, USA) following the manufacturer’s instructions, as described earlier (Singh et al., 2015).

### Data analysis

Genetic data analysis was performed using standard methods as described earlier (Hanekom et al., 2008; Narayanan et al., 2008), wherein spoligotypes were identified and analyzed as character types. The obtained spoligotyping patterns were compared with those available in the SITVIT_WEB database (http://www.pasteur-guadeloupe.fr:8081/SITVIT2). The Hunter-Gaston discriminatory index (HGDI) was used as a numerical index for MIRU-VNTR discriminatory power (Hunter and Gaston, 1988). The allelic diversity of the loci was identified as highly discriminant if the HGI was >0.6, moderately discriminant if the HGI was between 0.3 and 0.6, and poorly discriminant if the HGI was less than 0.3.

Differences among the lineages of the isolates and drug susceptibility patterns were analyzed by the chi-squared test and Fisher’s exact test as appropriate using STATA 9.0. The adjusted odds ratio (OR) and 95 percent confidence interval (CI) were calculated. Statistical significance was defined as a *p* value of 0.05 or less.

## Results

### Patient population and *M. tuberculosis* isolates

A total of 503 subjects who were suspected to have PTB and/or EPTB, were recruited from the Antiretroviral Therapy (ART) center and OPDs and wards of NITRD, New Delhi. Most of the patients were residents of Delhi (n=215, 74.9%), and the rest were from neighboring states in India, including Haryana (29, 10.1%), Uttar Pradesh (27, 9.4%), and Bihar (16, 5.6%), as these patients had visited the NITRD, New Delhi for their diagnosis and treatment **(** **Table 1** **)**.

**Table 1:** Demographic details of the patients, drug resistance patterns, and phylogenetic lineages of the MTB isolates.

Characteristics	Total N(%)	HIV negative	HIV positive	OR^*^ (95%CI)	p-value
**Gender** Female Male	84 (29.3) 203 (70.7)	59 (37.8) 97 (62.2)	25 (19.1) 106 (80.9)	1 2.6 (1.5-4.4)	<0.001
**Age group** <18 19-45 ≥46	30 (10.5) 194 (67.6) 63 (21.9)	26 (16.7) 95 (60.9) 35 (22.4)	4 (3.1) 99 (75.6) 28 (21.3)	1 6.8 (2.3-20.1) 5.2 (1.6-16.7)	<0.001 <0.006
**State of Residence** New Delhi Uttar Pradesh Haryana Bihar	215 (74.9) 27 (9.4) 29 (10.1) 16 (5.6)	111 (73.1) 22 (14.1) 10 (6.4) 10 (6.4)	101 (77.1) 5 (3.8) 19 (14.5) 6 (4.6)	1 0.3 (0.1-0.7) 2.1 (0.9-4.8) 0.6 (0.2-1.9)	<0.008 <0.05 <0.46
**Qualifications** Illiterate <10 10^th^ pass Graduate and above	44 (37.9) 24 (20.1) 27 (23.3) 21 (18.1)	22 (45.8) 3 (6.3) 13 (27.1) 10 (20.8)	22 (32.3) 21 (30.1) 14 (20.6) 11 (16.2)	1 7 (1.8-26.9) 1.1 (0.4-2.8) 1.1 (0.4-3.1)	<0.005 <0.88 <0.858
**Profession** Housewife Unemployed Others	21 (21.9) 21 (21.9) 54 (56.2)	17 (30.4) 11 (19.6) 28 (50.0)	4 (10.0) 10 (25.0) 26 (65.0)	1 3.8 (0.9-15.4) 3.9 (1.1-13.3)	<0.056 <0.027
**Marital Status** Married Unmarried Others**	202 (70.4) 63 (22.0) 22 (7.6)	116 (74.4) 40 (25.7) -	86 (65.7) 23 (17.5) 22 (16.8)	1 0.8 (0.4-1.4) -	<0.001
P/H/ATT^*^ No Yes	208 (72.5) 79 (27.5)	116 (74.4) 40(25.6)	92 (70.2) 39 (29.8)		<0.435
DST Patterns^*^ SIRE Sensitive MDR Others (Mono, Dual, Poly)	206 (74.6) 46 (16.7) 24 (8.7)	130 (83.3) 16(10.3) 10 (6.4)	76 (63.3) 30 (25.0) 14 (11.7)		<0.001
**Lineages** Beijing CAS EAI Others (Manu, LAM, T, U, X, Cameroon)	44 (15.9) 153 (55.4) 25 (9.1) 54 (19.6)	17 (10.9) 97 (62.2) 12 (7.7) 30 (19.2)	27 (22.5) 56 (46.7) 13 (10.8) 24 (20.0)		<0.024

*OR, Odds Ratio; P/H/ATT, Past History of Anti-Tuberculous Treatment; DST, Drug Susceptibility Testing; SIRE Sensitive, Sensitive for all four drugs (Streptomycin, Isoniazid, Rifampin, and Ethambutol); MDR, Multiple Drug Resistance.

** Others= widows/widowers, divorced or transgender.

These 503 patients were divided into two groups based on their HIV status. In the HIV/AIDS positive group, there were 249 of 503 (49.5%) patients who had HIV-TB coinfection, either PTB and/or EPTB. Their mean age was 35.6 ± 11.8 years. Of these, 130 (52.2%) subjects were mycobacterial culture positive and their mean age was 36.6 ± 12.5 years. In the HIV-negative group, we had 254 of 503 (50.5%) patients who had PTB and or EPTB. Their mean age was 32.8 ± 13.8 years. Of these patients, 156 (61.4%) were found to be mycobacterial culture positive with a mean age of 33.0 ± 14.0 years. This difference was statistically insignificant. Most patients were in the age group of 19-45 years with a *p*-value <0.001.

Of the 249 HIV seropositive patients, 220 (88.4%) were suspected of PTB and 29 (11.6%) were EPTB cases, while in 254 were HIV seronegative patients, 244 (96.1%) were PTB suspected, and the remaining 10 (3.9%) were EPTB suspected cases. Thus the prevalence of EPTB in HIV positive and HIV-negative was statistically highly significant (**Table 1**).

Overall, 287 patients were culture positive patients, of them 203 (70.7%) were male and 84 (29.3%) female. We found that male preponderance was common in both HIV-negative (62.2%) as well as in HIV-positive (80.9%) patients. However, this male preponderance was significantly (*p <*0.001) more in HIV-positive cases. All mycobacterial cultures were subjected to m-PCR to differentiate MTB and non-tuberculous mycobacteria (NTM). Samples from 11 (3.8%) patients were found to be NTM (all HIV positive) and the remaining 276 were MTB and subjected to genotyping studies (**Table 2**). Only one HIV-negative patient had NTM while in 10 HIV-positive patients (7.6%) NTM were isolated.

**Table 2:** Lineage distribution in pulmonary and extrapulmonary tuberculosis.

Total Subjects: 503					
MGIT Culture Positive: 287					
Confirmed as *M. tuberculosis* by species specific *multiplex PCR and subjected to genotyping:* 276					
HIV Status		Positive (n=120)		Negative (n=156)	
TB Type		PTB	EPTB	PTB	EPTB
Total		109	11	154	2
Lineages	CAS	51	5	97	0
	LAM	6	0	0	0
	BEIJING	25	2	17	0
	T	7	0	18	1
	U	7	0	0	0
	EAI	10	3	12	0
	Cameroon	1	0	1	1
	MANU	0	1	8	0
	X	2	0	0	0
	*M.africanum*	0	0	1	0

The socio-economic and educational data revealed that in the HIV positive group, most of the patients were literate but not matriculate. Patients in the HIV-TB group were likely to be unemployed or have their own small businesses. Of the patients, 202 (70.4%) were married while 63 (22.0%) were unmarried. The remaining 22 (7.65) were widows/widowers, divorcees, or transgender.

A history (6 months or more) of taking anti-tuberculous treatment (ATT) showed that 208 out of 287 (72.5%) of all the mycobacterial culture positive patients had a previous history of ATT. The probability of having a past history of tuberculosis was similar irrespective of HIV status. **(** **Table 1** **)**.

Before drug susceptibility testing and genotyping, all the culture isolates were checked by in-house multiplex PCR for species identification. Out of 287 isolates, 276 (96.2%) were identified as MTB, and the remaining 11 (3.8%) were identified as NTM. Ten (90.9%) NTM were isolated from HIV-positive patients and one (9.1%) from a HIV-negative patient.

Among 276 MTB isolates, 206 (74.6%) were sensitive to first line drugs and 46 (16.7%) were MDR. The remaining 24 (8.7%) were mono and dual resistant. Of the HIV-negative patients, 130 (83.3%) were SIRE sensitive while in HIV-positive cases, 76 (63.3%) were sensitive to first line drugs. Of the HIV-negative patients, 16 (10.3%) MDR cases were observed, while 30 (25.0%) MDR cases were observed in HIV-positive patients. The p-value was <0.001 **(** **Table 1** **)**.

Of the 276 culture positive MTB isolates, 120 were HIV-positive and 156 were HIV-negative. The lineage distribution of these populations in pulmonary and extrapulmonary tuberculosis infection is illustrated in **Table 2**.

The molecular characterization of the 276 MTB isolates revealed that the Central Asian Strain (CAS) genotype was found to be most predominant [153 (55.4%)], followed by Beijing lineage [44 (15.9%)], East African Indian [25 (9.1%)], and other lineages [54 (19.6%)]. In HIV-negative patients, the CAS genotype was predominant (62.2%) followed by Beijing (10.9%), EAI (7.7%), and others (19.25%). The distribution of various genotypes in HIV-positive patients was not significantly different and CAS genotypes remained predominant (**Tables 1**, **2**).

**Figure 1:**
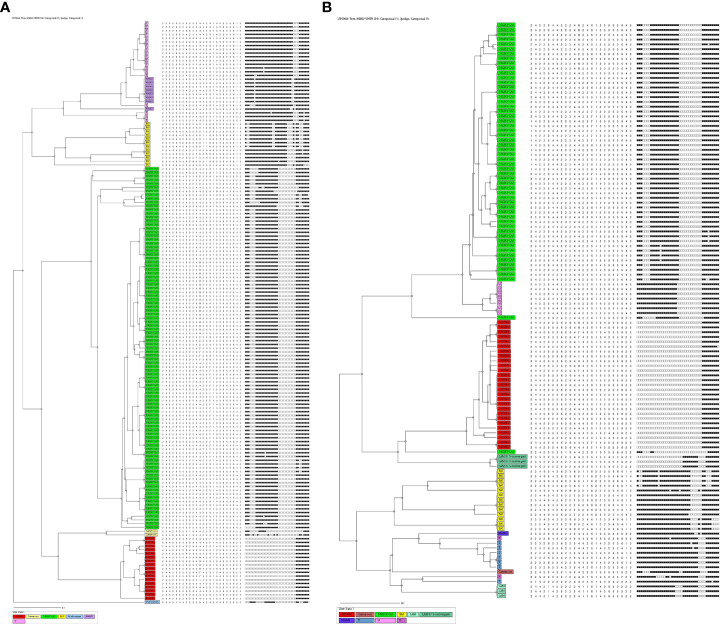
**(A)** UPGMA-dendrogram representing MIRU-VNTR and spoligotype patterns of 156 isolates from HIV-negative cases. **(B)** UPGMA-dendrogram representing MIRU-VNTR and spoligotype patterns of 120 isolates from HIV Positive cases.

Based on the spoligotyping pattern and 24 loci MIRU-VNTR analysis, an Un-weighted pair group method with arithmetic mean (UPGMA) phylogenetic tree was generated **(** **Figures 1A, B** **)** using MIRU-VNTR Plus online tool. Accordingly, in HIV-negative patients, a total of 19 STs comprising 140 (89.7%) isolates were identified while the remaining 16 (10.3%) isolates had unique/unidentified ST patterns. The lineages and corresponding spoligotype patterns of 156 isolates are shown in **Supplementary Table 1**. Of the 19 different STs observed, ST26 of CAS1_DEL had a maximum of 51.3% strains, followed by ST1 of Beijing with 10.9%, ST25 of CAS1_DEL with 3.2%, ST22 with 0.6%, ST53 of T1 with 7.7%, EAI3_IND with 4.5% and the remaining belonged to other STs. While in the 120 HIV-positive patients, the CAS family consisted of six STs (**Supplementary Table 2**). Among the STs of the CAS family, 37.5% of strains belonged to ST26, 3.3% each belonged to ST289 and ST1343, while 0.8% belonged to ST25 and ST2419 each.

In the EAI lineage, a total of 3 different STs were identified comprising 8 isolates of EAI, in which ST458 was found in 4 (3.3%) isolates followed by ST138 in 3 (2.5%) and ST1970 in 1 (0.8%) isolate. The single most dominant ST of the Beijing family was ST1 in 27 (22.5%) strains. Whereas other strains belonged to less predominant STs (**Supplementary Table 2).**

The distribution of the lineages in HIV positive and HIV negative patients were analyzed using the chi-square test which showed a significant (p=0.00816) difference in lineage distribution that was observed in both the study population (**Table 3**).

**Table 3:** Distribution of TB lineages in HIV positive and HIV negative population.

HIV status	TB-Lineages						P value
	CAS	EAI	Beijing	MANU	T	Cameroon	
Negative	97	12	17	8	19	2	0.00816
Positive	56	13	27	1	7	1

(Chi Square=15.576, df=5, p<0.05).

The combination of different epidemiological and phylogeographical techniques is important for better interpretation of molecular results. When the clustering rate by spoligotyping was determined, we found that Beijing was the most (100%) clustered lineage followed by CAS with 96.7% (89/92) in HIV-negative and 94.6% (53/56) in HIV-positive patients. The EAI lineage showed 87.5% clustering in HIV-negative while in HIV-positive patients it showed a 75% clustering rate. In HIV-negative cases, Manu was predominant with an 87.5% (7/8) clustering rate, followed by T lineage with 88.9% (16/18).

MIRU-VNTR revealed that 90.7% (88/97) and 96.4% (54/56) isolates of the largest cluster belonged to the CAS family in HIV-negative and HIV-positive patients respectively. In Beijing lineage, the clustering rate was 70.6% in HIV-negative and 63% in HIV-positive cases which was much lower than spoligotyping. Using MIRU-VNTR we identified 131 different genotypes in 34 clusters and 25 orphan isolates with a clustering rate of 84% in HIV negative cases, whereas in HIV positive cases 101 various genotypes were recognized in 28 clusters with an 84.2% clustering rate (**Table 4**).

**Table 4:** Distribution and clustering patterns of various lineages of *M. tuberculosis* in HIV seronegative and HIV seropositive patients.

Lineage	HIV status	Method of typing	No. of isolates	No. of clusters (size)	No. of clustered strains (%)	No. of orphan strains (%)	No. of unique strains
**CAS**	**HIV Neg**	Spoligotyping MIRU-VNTR	92 97	4 (2-80) 21 (2-8)	89 (96.7) 88 (90.7)	3 (3.3) 9 (9.3)	0 0
	**HIV Pos**	Spoligotyping MIRU-VNTR	56 56	3 (4-45) 15 (2-6)	53 (94.6) 54 (96.4)	3 (5.4) 2 (3.6)	0 0
**EAI**	**HIV Neg**	Spoligotyping MIRU-VNTR	8 12	1 (7) 3 (2-3)	7 (87.5) 8 (66.7)	1 (12.5) 4 (33.3)	0 0
	**HIV Pos**	Spoligotyping MIRU-VNTR	4 13	1 (3) 3 (3-4)	3 (75) 10 (76.9)	1 (25) 3 (23.1)	0 0
**Beijing**	**HIV Neg**	Spoligotyping MIRU-VNTR	17 17	1 (17) 4 (2-4)	17 (100) 12 (70.6)	0 5 (29.4)	0 0
	**HIV Pos**	Spoligotyping MIRU-VNTR	27 27	1 (27) 5 (2-8)	27 (100) 17 (63)	0 10 (37)	0 0
**Cameroon**	**HIV Neg**	Spoligotyping MIRU-VNTR	0 2	0 0	0 0	0 2 (100)	0 0
**LAM**	**HIV Pos**	Spoligotyping MIRU-VNTR	6 6	2 (3) 2 (2-3)	6 (100) 5 (83.3)	0 1 (16.7)	0 0
**MANU**	**HIV Neg**	Spoligotyping MIRU-VNTR	8 8	1 (7) 1 (7)	7 (87.5) 7 (87.5)	1 (12.5) 1 (12.5)	0 0
	**HIV Pos**	Spoligotyping MIRU-VNTR	1 1	0 0	0 0	1 (100) 1 (100)	0 0
**T**	**HIV Neg**	Spoligotyping MIRU-VNTR	18 19	3 (2-12) 5 (2-5)	16 (88.9) 16 (84.2)	2 (11.1) 3 (15.8)	0 0
	**HIV Pos**	Spoligotyping MIRU-VNTR	7 7	4 (4) 2 (2-3)	4 (57.1) 5 (71.4)	3 (42.9) 2 (28.6)	0 0
**X**	**HIV Pos**	Spoligotyping MIRU-VNTR	2 2	0 0	0 0	2 (100) 2 (100)	0 0
**U**	**HIV Pos**	Spoligotyping MIRU-VNTR	7 7	1 (7) 2 (2-4)	7 (100) 6 (85.7)	0 1 (14.3)	0 0
**M. africanum**	**HIV Neg**	Spoligotyping MIRU-VNTR	0 1	0 0	0 0	0 1 (100)	0 0
**UK**	**HIV Neg**	Spoligotyping MIRU-VNTR	13 0	1 (2) 0	2 (15.4) 0	3 (23.1) 0	8 (61.5) 0
	**HIV Pos**	Spoligotyping MIRU-VNTR	10 0	1 (4) 0	4 (40) 0	2 (20) 0	4 (40) 0
**Total**	**HIV Neg**	Spoligotyping MIRU-VNTR	156 156	11 (2-80) 34 (2-8)	138 (88.5) 131 (84)	10 (6.4) 25 (16)	8 (5.1) 0
	**HIV Pos**	Spoligotyping MIRU-VNTR	120 120	11 (3-45) 28 (2-8)	108 (90) 101 (84.2)	12 (10) 19 (15.8)	0 0

**Figure 2:**
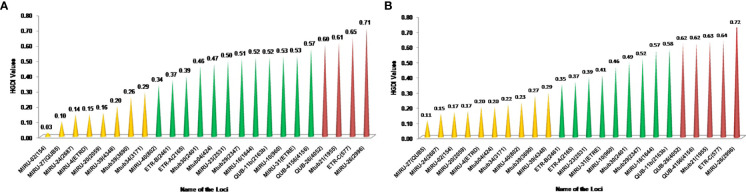
**(A)** Minimum spanning trees of MTC isolates from HIV-negative patients typed with both spoligotyping and MIRU-VNTR. Each circle represents a genotype. The distance between circles represents how closely related the different genotypes are to each other. MST connects each genotype based on the degree of changes required to go from one allele to another. The configuration of the tree is represented by branches *i.e*. continuous versus dotted lines and circles representing each pattern. The length of the branches denotes the distance between patterns whereas the intricacy of the lines indicates the number of spacers altered between two patterns. The thicker lines represent one change while thinner ones indicate 2 or 3. The size of the circle is comparable to the total number of MTB isolates in this study. The color of the circles represents the phylogenetic lineage to which the specific pattern belongs. Beijing patterns are circled in red. Patterns colored in green indicate Delhi/CAS strains. EAI strains are depicted in yellow. **(B)** Minimum spanning trees of MTC isolates from HIV-positive patients typed with both spoligotyping and MIRU-VNTR. Each circle represents a genotype. The distance between circles represents how closely related the different genotypes are to each other (detailed description as mentioned in **Figure 3A**).

In HIV-negative cases, the largest and smallest cluster comprised 80 and 2 isolates respectively by spoligotyping. However, in HIV-positive cases, the largest cluster consisted of 45 isolates, while the smallest cluster had 3 isolates. When orphan strains were checked by both methods in all lineages, we found 6.4% (10/156) of orphan strains by spoligotyping in HIV-negative cases, while using MIRU-VNTR, the rate was 16% (25/156). In HIV-positive cases, spoligotyping showed 10% (12/120) isolates as an orphan while MIRU-VNTR revealed 15.8% (19/120) (**Table 5**). We did not find any significant difference with regard to the demographic background of the patients as most of them were from North India only (**Table 6**). The results of 24 -loci MIRU-VNTRs analysis showed that loci QUB-26, Mtub21, ETR-c, and MIRU-26 were highly discriminant in MTB isolates from HIV-negative as well as HIV-positive cases (**Figures 2A, B**).

**Table 5:** Overall clustering patterns of MTB isolates by both the molecular techniques in HIV-negative and HIV-positive patients.

Genotyping methods and type of patients	No. of isolates	No. of clusters (size)	No. of clustered strains (%)	No. of orphan strains (%)	No. of unique strains (%)
Spoligotyping					
HIV Negative	156	11 (2-80)	138 (88.5)	10 (6.4)	8 (5.0)
HIV Positive	120	11 (3-45)	108 (90.0)	12 (10.0)	–
MIRU-VNTR					
HIV Negative	156	34 (2-8)	131 (84.0)	25 (37)	0
HIV Positive	120	28 (2-8)	101 (84.2)	19 (15.8)	0

**Table 6:** Co-relation of various demographical and other factors between Clustered and Unique MTB strains.

Characteristics	Total N (%)	Genotyping patterns		OR (95%CI)	p-value
		Unique	Clustered		
**Gender** Female Male	84 (30.4) 192 (69.6)	10 (30.3) 23 (69.7)	74 (30.5) 169 (69.5)	1 0.9 (0.5-2.1)	0.986
**Age group** <18 19-45 ≥46	29 (10.5) 187 (67.6) 60 (21.7)	3 (9.1) 25 (75.7) 5 (15.2)	26 (10.6) 187 (67.7) 60 (21.7)	1 0.7 (0.2-2.7) 1.2 (0.3-5.7)	0.653 0.756
**HIV Status** HIV Negative HIV Positive	156 (56.5) 120 (43.5)	14 (42.4) 19 (57.6)	142 (58.4) 101 (41.6)	1 0.5 (0.3-1.1)	0.085
**State of Residence** New Delhi Uttar Pradesh Haryana Bihar	206 (74.6) 26 (9.4) 28 (10.1) 16 (5.8)	22 (66.7) 3 (9.1) 6 (18.1) 2 (6.1)	184 (75.7) 23 (9.4) 28 (10.1) 16 (5.8)	1 0.9 (0.3-3.3) 0.4 (0.2-1.1) 0.8 (0.2-3.9)	0.894 0.108 0.821
**Qualifications** Illiterate <10 10^th^ pass Graduate and above	44 (40.0) 21 (19.1) 27 (24.5) 18 (16.4)	5 (38.5) 2 (15.4) 4 (30.8) 2 (15.3)	39 (40.2) 19 (19.6) 23 (23.7) 16 (16.5)	1 1.2 (0.2-6.9) 0.7 (0.2-3.0) 1.0 (0.2-5.8)	0.823 0.672 0.977
**Profession** Housewife Unemployed Others	21 (23.1) 21 (23.1) 49 (53.9)	4 (26.7) 6 (40.0) 5 (33.3)	17 (22.4) 15 (19.7) 44 (57.9)	1 0.6 (0.1-2.5) 2.1 (0.5-8.6)	0.471 0.318
**Marital Status** Married Unmarried Others	196 (71.0) 63 (22.8) 17 (6.2)	18 (54.5) 13 (39.4) 2 (6.1)	178 (73.3) 50 (20.6) 15 (6.1)	1 0.4 (0.2-0.8) 0.8 (0.2-3.6)	<0.01 0.727
**P/H/ATT** No Yes	58 (39.2) 90 (60.8)	10 (50.0) 10 (50.0)	48 (37.5) 80 (62.5)	1 1.7 (0.6-4.3)	0.290
**DST Patterns** SIRE Sensitive MDR Others (Mono, dual etc)	206 (74.6) 46 (16.7) 24 (8.7)	24 (72.7) 5 (15.2) 4 (12.1)	182 (74.9) 41 (16.9) 20 (8.2)	1 1.1 (0.4-3.0) 0.6 (0.2-2.1)	0.881 0.480
**Lineages** Beijing CAS EAI Others (Manu, LAM, T, U, X, Cameroon)	44 (15.9) 153 (55.4) 25 (9.1) 54 (19.6)	9 (27.3) 6 (18.2) 5 (15.2) 13 (39.4)	35 (14.4) 147 (60.5) 20 (8.2) 41 (16.9)	1 6.2 (2.1-18.9) 1.0 (0.3-3.5) 0.8 (0.3-2.1)	<0.001 0.964 0.670

The minimum Spanning Tree (MST) analysis was done by using MIRU-VNTRplus. The various SITs amongst MTB isolates from HIV-negative patients (**Figure 2A**) and HIV-positive patients (**Figure 2B**) showed predominant SITs and the evolutionary relationship between the lineages and their SITs.

## Discussion

This study describes the genetic diversity in *M. tuberculosis* isolated from HIV-TB co-infected and HIV uninfected TB cases. Most of our patient population was from the North Indian states of Delhi’s national capital region, Haryana and Uttar Pradesh. Therefore, the incidence and prevalence rates of HIV and TB were in line with other studies. However, in our study, we compared the drug resistance pattern and genotypes side-by-side isolated from these two patient groups under a controlled single laboratory study.

As reported in various other studies, we found that CAS was the predominant genotype followed by Beijing and EAI (Devi et al., 2015; Singh et al., 2015; Singh et al., 2021). Interestingly, the same pattern was seen in our patient populations irrespective of their HIV status. This could be explained based on the circulation of these genotypes in the environment and the ethnic make-up of North Indian patients (Sankar et al., 2013). In our earlier studies, we also found similar patterns in various genotypes (Sankar et al., 2013; Singh et al., 2015). Similar results were also found in the southern part of India (Narayanan et al., 2008). However, in this present study, the Beijing genotype was more prevalent in HIV seropositive patients rather than HIV-negative patients (**Table 1**). Similar results were also reported in a study conducted on HIV-positive patients with TB meningitis (Caws et al., 2006). Narayanan et al. (Narayanan et al., 2008) and Gupta et al. (2014) also reported Beijing genotype only in HIV-infected patients and not in HIV-uninfected patients. et alVeigas et al. (Viegas et al., 2013) suggested that the association of Beijing strain with HIV seropositive status could be due to the combination of the increased virulence of the strain, and higher susceptibility of HIV positive patients. However, a study from Western Maharashtra, India found no association between HIV status and spoligotypes (Chatterjee et al., 2010).

Generally, the prevalence of different genotypes of MTB is dependent on the geographical location and the ethnicities of the population, as reported in our previous studies (Sankar et al., 2013; Singh et al., 2015). In the present study, Beijing genotypes were also significantly more common in HIV-positive patients and CAS lineage was more common in HIV-negative cases, which could be explained by the fact that CAS lineage is the most common lineage circulating in northern parts of India (Sankar et al., 2013), and the same pattern was seen in this study. A population-based investigation for 20 years in rural Malawi reported ST129 (EAI5) to be the most common lineage associated with HIV status (Glynn et al., 2010). A study from Ethiopia found that in HIV-positive subjects, the T family was the most predominant (38.5%) (Mihret et al., 2012). This also suggests that the frequency of various lineages depends more on geographical locations rather than HIV status.

Anti-TB drug resistance can develop after acquiring the infection (secondary), but it can also be a baseline (primary) when the person is infected with drug-resistant strains of *M. tuberculosis*. In the present study, we observed an overall drug resistance (resistance to any drug) in 25.4% and MDR in 16.7% isolates (**Table 1**). A higher rate of MDR was found in isolates from HIV-positive patients as compared to isolates from HIV-negative patients. This may be attributed to the previous history of TB in a higher number of patients from the HIV-positive group, as well as the lower socio-economic status of these patients. Saldanha et al. (2019) also reported a high prevalence of MDR-TB in HIV-positive patients and associated this with the treatment of previous TB infection with sub-optimal anti-TB regimens. Similarly, Sethi et al. (2013) reported that TB was more prevalent in patients from lower socioeconomic backgrounds and that there was a higher number of MDR-MTB (27.3%) in HIV seropositive subjects compared to HIV seronegative subjects (15.4%). A prior study carried out by Isaakidis et al. (2014) also confirmed the very high burden of DR-TB in HIV-positive patients (38%) at an ART center in Mumbai. In the current study, most of our patients were from Delhi and its adjoining states. Our findings may not necessarily represent the entire country, as the socio-economic conditions of patients, HIV prevalence, and medical practices differ significantly from region to region. Clustering is often used to group strains with similar genotypic traits. On the analysis of clustering amongst the various phylogenetic lineages, we found clustering in 88.8% and 86.6% HIV-uninfected and HIV-infected patient groups. This reflects the high transmission rate within these population groups (Odhiambo et al., 1999). In addition to sub-lineage analysis, we also observed that CAS1_DEL (ST26) and Beijing (ST1) clades were predominant in both the study populations with no significant difference. These findings are in line with previous publications (Singh et al., 2004; Arora et al., 2009; Sankar et al., 2013; Singh et al., 2015).

Nutrition and timely medical care are important determinants of disease outcome. In India, even though TB is more prevalent in male patients and a poor disease outcome is more likely in females due to various socio-economic factors (Singh et al., 2012) and the finding of our study were on similar lines.

There was no significant difference in the prevalence of clustered or unique isolates among different age groups. The percentage of clustered isolates based on 24 loci MIRU-VNTR was, however, higher among married patients. This could be due to better opportunities for the circulation of these strains in the families, though it may an overestimation (García de Viedma et al., 2011). We found a higher percentage of clustered isolates of CAS lineage which showed a significantly higher clustering rate than other lineages. This may be because CAS is more prevalent in North India and most of the isolates from both groups are from north India (Arora et al., 2009; Singh et al., 2015). We also observed unique isolates with lineages like Manu, LAM, T, U X, and Cameroon, demonstrating an assortment of strains and newly emerging strains in these patients, and that this trend should be considered seriously by the program managers.

**Figure 3:**
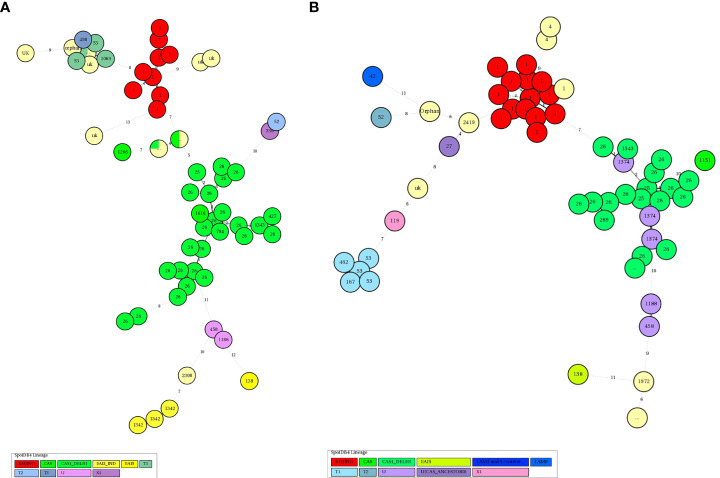
**(A)** The Hunter-Gaston discriminatory index of MIRU-VNTR alleles of TB isolates from HIV-Negative patients MIRU: Mycobacterial Interspersed Repetitive Units, VNTR, Variable Number Tandem Repeats; HGDI, Hunter-Gaston Diversity index. The allelic diversity of the loci was classified as highly discriminant [Hunter-Gaston Index **(HGI)** >0.6], moderately discriminant (HGI ≥0.3 to ≤0.6), and poorly discriminant (HGI < 0.3). **(B)** The Hunter-Gaston discriminatory index of MIRU-VNTR alleles of TB isolates from HIV- TB co-infected patients.

As per our findings in the HIV-negative group as well as in those in the HIV-positive group, MIRU26, ETRC, Mtub21, QUB4156, and QUB26 were highly discriminating, but MIRU2, MIRU24, MIRU20, and ETRD were feebly discriminating (**Figures 3A, B**). Earlier we also found that QUB4156, QUB26, and MIRU26 were highly discriminating and MIRU2, MIRU4 or ETRD and MIRU24 were poorly discriminating (Sankar et al., 2013).

It might be expected that some lineages and clones of MTB are more prevalent or have severe disease outcomes in HIV-positive patients. However, in the present study, we found that though all clones could be isolated from HIV-positive patients, the incidence of the Beijing genotype was more frequent in this group of patients. It is important to note that in North India, even though the CAS lineage is more prevalent, in HIV-infected patients CAS lineage had a low incidence compared to Beijing lineage. We are not able to explain fully why the EPTB in the HIV-infected patients in our study was not as common as that reported by others (Yang et al., 1995; Giri et al., 2013), but it was significantly more common (11.6%) than in HIV-negative patients (3.9%). This could be because our patient recruiting center is a dedicated TB hospital and EPTB cases are more commonly referred to other general care hospitals for want of wider scope for surgical and medical management.

## Conclusions

The present study provides important molecular genotypic data of *M. tuberculosis* isolates from HIV-negative and HIV-positive patients of Northern India. Central Asian lineage despite being the most predominant isolate circulating in the study area and both HIV-positive and HIV-negative TB cases, the Beijing genotype showed a statistically significant high incidence rate in HIV-TB cases. However, the clustering rate of predominant genotypes was similar in both HIV-positive and HIV-negative TB cases, which shows the high transmissibility of these genotypes in the community. Probability of having extrapulmonary tuberculosis and isolation of non-tuberculous mycobacterial were also significantly more in HIV-infected patients.

## Data availability statement

The original contributions presented in the study are included in the article/**Supplementary Material**. Further inquiries can be directed to the corresponding author.

## Ethics statement

The studies involving human participants were reviewed and approved by Institutional Ethical Committee, All India Institute of Medical Sciences, New Delhi - 110029. Written informed consent to participate in this study was provided by the participants’ legal guardian/next of kin.

## Author contributions

SS, NS, DB, and UA designed the study. JS, NS, RS, and GS collected clinical samples and patient clinical information. JS performed genotyping analysis. JS, RS, and GS performed the experiments. SS and JS analysed data. JS, NS, and SS wrote the manuscript. Final modifications in manuscript done by MM and AM. SS arranged for financial support. All authors contributed to the article and approved the submitted version.

## Funding

The study received financial support from Indian Council of Medical Research (ICMR), New Delhi (ICMR) Sanction Order Number: 5/8/5/15/10-ECD-I) and publication support from Indo-U.S. Science and Technology Forum (IUSSTF) (Award Letter No. IUSSTF/USISTEF/8th Call/HI-051/2017/2018-19).

## Acknowledgments

We are thankful to all the patients who cooperated in this study. We are also grateful to Indian Council of Medical Research (ICMR), New Delhi (ICMR Sanction Order Number: 5/8/5/15/10-ECD-I) and Indo-U.S. Science and Technology Forum (IUSSTF) (Award Letter No. IUSSTF/USISTEF/8th Call/HI-051/2017/2018-19) for financial support.

## Conflict of interest

The authors declare that the research was conducted in the absence of any commercial or financial relationships that could be construed as a potential conflict of interest.

## Publisher’s note

All claims expressed in this article are solely those of the authors and do not necessarily represent those of their affiliated organizations, or those of the publisher, the editors and the reviewers. Any product that may be evaluated in this article, or claim that may be made by its manufacturer, is not guaranteed or endorsed by the publisher.

## Abbreviation

TB, Tuberculosis; PTB, Pulmonary Tuberculosis; EPTB, Extrapulmonary Tuberculosis; HIV, Human Immunodeficiency Virus; MIRU-VNTR, Mycobacterial Interspersed Repetitive Unite - Variable Number Tandem Repeat; DST-SIRE, Drug Susceptibility Testing for Streptomycin (STR), Isoniazid (INH), Rifampin (RIF), and Ethambutol (EMB); CAS, Central Asian; EAI, East-African-Indian.
