# Supplementary material for: A comparative analysis of molecular genotypes of Mycobacterium tuberculosis isolates from HIV-positive and HIV-negative patients
Source: Front Cell Infect Microbiol. 2022 Oct 12;12:953443. doi: 10.3389/fcimb.2022.953443 (PMC9597297; doi:10.3389/fcimb.2022.953443)
Supplement: Supplementary file 1 [file Table_1.docx]

**Supplementary Table 1** Spoligotype share type of *M. tuberculosis* strains from HIV negative patients (n=156)

| **S.no.** | **SIT*** | **Spoligotype pattern** | **Octal code** | **Lineage ǂ** | **No. of isolates in this study (%)** | **Clustered or Unique**  **SIT #** |
| --- | --- | --- | --- | --- | --- | --- |
| 1 | 1 | □□□□□□□□□□□□□□□□□□□□□□□□□□□□□□□□□□■■■■■■■■■ | 000000000003771 | BEIJING | 17(10.9) | Clustered |
| 2 | 22 | ■■■□□□□■■■■■■■■■■■■□□□□□□□□□□□□□□□□■■■■■■■■ | 703777400001771 | CAS | 1(0.6) | Unique |
| 3 | 25 | ■■■□□□□■■■■■■■■■■■■■■■□□□□□□□□□□□□■■□□■■■■■ | 703777740003171 | CAS1_DEL | 5(3.2) | Clustered |
| 4 | 26 | ■■■□□□□■■■■■■■■■■■■■■■□□□□□□□□□□□□■■■■■■■■■ | 703777740003771 | CAS1_DEL | 80(51.3) | Clustered |
| 5 | 52 | ■■■■■■■■■■■■■■■■■■■■■■■■■■■■■■■■□□□□■■■□■■■ | 777777777760731 | T2 | 2(1.3) | Clustered |
| 6 | 53 | ■■■■■■■■■■■■■■■■■■■■■■■■■■■■■■■■□□□□■■■■■■■ | 777777777760771 | T1 | 12(7.7) | Clustered |
| 7 | 138 | ■■■■■■■■■■■■■■■■■■■■■■■■■■■■□□□□■□■■■■■□□□□ | 777777777413700 | EAI5 | 1(0.6) | Unique |
| 8 | 336 | ■■■■■■■■■■■■■■■■■□■■■■■■■■■■■■■■□□□□■■■□■■■ | 777776777760731 | T2 | 1(0.6) | Unique |
| 9 | 427 | ■■■□□□□■■■■■□□□■■■■■■■□□□□□□□□□□□□■■■■■■■■■ | 703707740003771 | CAS1_DEL | 1(0.6) | Unique |
| 10 | 458 | ■■■■■■■■■■■■■■■■■■■■■■■■■■■■□□□□□□■■■■■■■■■ | 777777777403771 | EAI5 | 1(0.6) | Unique |
| 11 | 498 | ■■■■■■■■■■■□■■■■■■■■■■■■■■■■■■■■□□□□■■■■■■■ | 777677777760771 | T1 | 2(1.3) | Clustered |
| 12 | 794 | ■■■□□□□■■■■■■□■■■■■■■■□□□□□□□□□□□□■■■■■■■■■ | 703757740003771 | CAS1_DEL | 2(1.3) | Clustered |
| 13 | 1069 | ■■■■■■□□■■■■■■■■■■■■■■■■■■■■■■■■□□□□■■■■■■■ | 771777777760771 | T1 | 1(0.6) | Unique |
| 14 | 1186 | ■■■■■■■■■■■■■■■■■■■■■■■■■■■■□□□□□□■■■■□■■■■ | 777777777403671 | UK | 2(1.3) | Clustered |
| 15 | 1266 | ■■■□□□□□□■■■■■■■■■■■■■□□□□□□□□□□□□■■■■■■■■■ | 700777740003771 | CAS | 2(1.3) | Clustered |
| 16 | 1342 | ■□□■■■■■■■■■■■■■■■□□■■■■■■■■□□□□■□■■□□□■■■■ | 477777177413071 | EAI3_IND | 7(4.5) | Clustered |
| 17 | 1343 | ■■■□□□□■■■■■□■■■■■■■■■□□□□□□□□□□□□■■■■■■■■■ | 703737740003771 | CAS1_DEL | 1(0.6) | Unique |
| 18 | 1616 | □□□□□□□■■■■■■■■■■■■■■■□□□□□□□□□□□□■■■■■■■■■ | 003777740003771 | UK | 1(0.6) | Unique |
| 19 | 2308 | ■■■■■■■■■■■■■■■■■■■■■■□■■■■■□□□□□□■■■■■■■■■ | 777777757403771 | UK | 1(0.6) | Unique |
| 20 | Orphan | ■■■■■■□■■■■■■■■■■■■■■■■■■■■■■■■■□□■■■■■■■■■ | 773777777763771 | MANU2 | 7(4.5) | Clustered |
| 21 | Uk | ■■■□□□□□□■■□□■■■■■■■■■□□□□□□□□□□□□□□■■■■■■■ | 700637740000771 | UK | 1(0.6) | Unique |
| 22 | Uk | ■■■□□□□□□■■■■■□□□□□□□□□□□□□□□□□□□□□□□□■■■■■ | 700760000000171 | UK | 1(0.6) | Unique |
| 23 | Uk | ■■■□□□□■■■■■■■■■■■■■■■□□□□□□□□□□□□■■□□■■■■□ | 703777740003170 | UK | 1(0.6) | Unique |
| 24 | Uk | ■■■□□□□■■■■■■■■■■■■■■■■■■■■■■■■■□□■■■■■■■■■ | 703777777763771 | UK | 1(0.6) | Unique |
| 25 | Uk | ■■■□□■□□■□□□□□□■□□□■□■□□□□□□□□□□□□□□■■□■■■■ | 711004240000671 | UK | 1(0.6) | Unique |
| 26 | Uk | ■■■■■■■■■■■■■■■■■■■■■□□□□□□□□□□□□□■■□□□■■■■ | 777777700003071 | UK | 1(0.6) | Unique |
| 27 | Uk | ■■■■■■■■■■■■■■■■■■■■■■■■■■■■■■□□■□■■■■■■■■■ | 777777777713771 | UK | 1(0.6) | Unique |
| 28 | Uk | □□□■■■■□□□□□□■■■■■■■□□□□□□□□□□□□□□■■□□□■■■□ | 074037600003070 | UK | 1(0.6) | Unique |
| 29 | Uk | ■■■□□□□□□□■■■■■■■■■■■■□□□□□□□□□□□□■■□□■■■■■ | 700377740003171 | UK | 1(0.6) | Unique |

*SIT: Shared International Type (SITVIT_WEB)

ǂClade designations according to SITVIT_WEB.

# Clustered strains correspond to a similar spoligotype pattern shared by 2 or more strains “within this study”; as opposed to unique strains harbouring a spoligotype pattern that does not match with another strain from this study. Whereas strains matching a pre-existing pattern in SITVIT2 database with single pattern are referred a “Unique” and those that does not match are designated as “orphan”.

**Supplementary Table 2** Spoligotype share type of *M. tuberculosis* strains from HIV Positive patients (n=120)

| **S.no** | **SIT*** | **Spoligotype pattern** | **Octal code** | **Lineage ǂ** | **No. of isolates in this study (%)** | **Clustered or Unique**  **SIT #** |
| --- | --- | --- | --- | --- | --- | --- |
| 1 | 1 | □□□□□□□□□□□□□□□□□□□□□□□□□□□□□□□□□□■■■■■■■■■ | 000000000003771 | BEIJING | 27(22.5) | Clustered |
| 2 | 4 | □□□□□□□□□□□□□□□□□□□□□□□□■■■■■■■■□□□□■■■■■■■ | 000000007760771 | LAM 3/S-convergent | 3(2.5) | Clustered |
| 3 | 25 | ■■■□□□□■■■■■■■■■■■■■■■□□□□□□□□□□□□■■□□■■■■■ | 703777740003171 | CAS1_DEL | 1(0.8) | Unique |
| 4 | 26 | ■■■□□□□■■■■■■■■■■■■■■■□□□□□□□□□□□□■■■■■■■■■ | 703777740003771 | CAS1_DEL | 45(37.5) | Clustered |
| 5 | 27 | ■■■□□□□■■■■■■■■■■■■■■■□□■■■■■■■■■□□□□■■■■■■ | 703777747770371 | UK | 1(0.8) | Unique |
| 6 | 42 | ■■■■■■■■■■■■■■■■■■■■□□□□■■■■■■■■□□□□■■■■■■■ | 777777607760771 | LAM9 | 3(2.5) | Clustered |
| 7 | 52 | ■■■■■■■■■■■■■■■■■■■■■■■■■■■■■■■■□□□□■■■□■■■ | 777777777760731 | T2 | 1(0.8) | Unique |
| 8 | 53 | ■■■■■■■■■■■■■■■■■■■■■■■■■■■■■■■■□□□□■■■■■■■ | 777777777760771 | T1 | 4(3.3) | Clustered |
| 9 | 119 | ■■□■■■■■■■■■■■■■■■■□□■■■■■■■□□□□■□■■■■■■■■■ | 777776777760771 | X1 | 1(0.8) | Unique |
| 10 | 138 | ■■■■■■■■■■■■■■■■■■■■■■■■■■■■□□□□■□■■■■■□□□□ | 777777777413700 | EAI5 | 3(2.5) | Clustered |
| 11 | 167 | ■■■■■■■■■■■■■■■■■■■■■■■■■■■■■□■■□□□□■■■■■■■ | 777777777660771 | T1 | 1(0.8) | Unique |
| 12 | 289 | ■■■□□□□■■■■■■■■■■■■■■■□□□□□□□□□□□□■■■□■■■■■ | 703777740003571 | CAS1_DEL | 4(3.3) | Clustered |
| 13 | 458 | ■■■■■■■■■■■■■■■■■■■■■■■■■■■■□□□□□□■■■■■■■■■ | 777777777403771 | EAI5 | 4(3.3) | Clustered |
| 14 | 462 | ■■■■■■■■■■■■■■■■■■■■■■■■■■■■□■■■□□□□■■■■■■■ | 777777777560771 | T1 | 1(0.8) | Unique |
| 15 | 1151 | ■■■□□□□□□□■■■■■■■■■■■■□□□□□□□□□□□□□■■■■■■■■ | 700377740001771 | CAS | 1(0.8) | Unique |
| 16 | 1188 | ■■■■■■■■■■■■■■■■■■■■■■■■■■■□□□□□□□■■■■■■■■■ | 777777777003771 | UK | 1(0.8) | Unique |
| 17 | 1343 | ■■■□□□□■■■■■□■■■■■■■■■□□□□□□□□□□□□■■■■■■■■■ | 703737740003771 | CAS1_DEL | 4(3.3) | Clustered |
| 18 | 1374 | ■■■■■■■■■■■■■■■■■■■■■□□□□□□□□□□□□□■■■■■■■■■ | 777777700003771 | U | 7(5.8) | Clustered |
| 19 | 1970 | ■■■■■■■■■■■■■■■■■□■■■■□■■■■■□□□□■□■■■■■■■■■ | 777776757413771 | EAI6-BGD1 | 1(0.8) | Unique |
| 20 | 2419 | ■■■□□□□■■□□□□□□□□□□□□□□□□□□□□□□□□□□□□■■■■■■ | 703000000000371 | CAS | 1(0.8) | Unique |
| 21 | Orphan | ■■■■■■■■■■□□□□□■■□■■■■■■■■■■■■■■□□□□■■■■■■■ | 777406777760771 | X1 | 1(0.8) | Unique |
| 22 | Uk | ■■■■■■■■■■■■■■■■■■■■■■■■■■■■■■□□■□■■■■■■■■■ | 777777777713771 | MANU1 | 4(3.3) | Clustered |
| 23 | Uk | ■□□■■■■■■■■□■■■■■■■■■■■■■■■■□□□□□□■■□□□■■■■ | 477677777403071 | UK | 1(0.8) | Unique |

*SIT: Shared International Type (SITVIT_WEB) ǂClade designations according to SITVIT_WEB.# Clustered strains correspond to a similar spoligotype pattern shared by 2 or more strains “within this study”; as opposed to unique strains harbouring a spoligotype pattern that does not match with another strain from this study. Whereas strains matching a pre-existing pattern in SITVIT2 database with single pattern are referred a “Unique” and those that does not match are designated as “orphan”.
